# The Thermal Influence of an Electromagnetic Field with a Radio Frequency Depending on the Type of Electrode Used

**DOI:** 10.3390/ijerph191811378

**Published:** 2022-09-09

**Authors:** Kamil Bryś, Beniamin Oskar Grabarek, Piotr Król, Rafał Staszkiewicz, Magdalena Wierzbik-Strońska, Tomasz Król

**Affiliations:** 1 Department of Histology, Cytophysiology and Embryology, Faculty of Medicine, Academia of Silesia in Katowice, 41-800 Zabrze, Poland; 2 Institute of Sport Sciences, Academy of Physical Education in Katowice, 40-065 Katowice, Poland; 3 5th Military Clinical Hospital with the SP ZOZ Polyclinic in Krakow, 30-901 Krakow, Poland; 4 Faculty of Medicine, Academia of Silesia in Katowice, 41-800 Zabrze, Poland; 5 Department of Kinesitherapy and Special Methods, School of Health Sciences in Katowice, Medical University of Silesia in Katowice, 40-055 Katowice, Poland

**Keywords:** diathermy, resistive electrode, capacitive electrode, tissue temperature, physiotherapy

## Abstract

Diathermy is a method used in physiotherapy based on obtaining an increase in temperature by supplying energy from the electromagnetic field to the tissues. The aim of this retrospective work, based on the data included in a medical documentation, was to assess the dynamics of temperature changes on the body surface after the application of a high-frequency electromagnetic field depending on the type of electrode used. In order to generate a radio frequency electromagnetic field, an INDIBA ACTIV^®^ CT9 was used. In order to measure the temperature, an HT-17 thermovision camera was used, enabling measurements within the range of −20 to 300 °C, with an accuracy of ±2% or 2 °C. The participants consisted of 30 healthy subjects (15 women and 15 men) who were physiotherapy students in the Faculty of Public Health in the Silesian Medical University in Katowice, Poland; they were divided into two comparative groups (A and B). It was found that the differences between the groups were not significant in the measurements carried out before using the electrode (*p* = 0.84; Mann–Whitney U test). On the other hand, at 0, 5 and 15 min, statistically significant differences were noted in the tissue temperature between the groups, depending on the electrode used (*p* = 0.00; Mann–Whitney U test). Based on the obtained results, it can be concluded that with the extension of the observation time, the tissue temperature increased (for Group A, Me 30.40 °C vs. 34.90 °C; for Group B, Me 30.70 °C vs. 35.20 °C). Our study confirmed that the use of both a capacitive and resistive electrode during treatment with the use of a high-frequency electromagnetic field statistically significantly increased the surface temperature of the area to which the therapy was applied. The results of the study can be used in clinical practice by physiotherapists to optimize the conditions of therapy.

## 1. Introduction

Despite there being relatively few scientific studies, work at low frequencies (<1 MHz) is used in clinical practice, with the goal of obtaining an increase in tissue temperature as well as other physiological effects. Among the electrophysical factors used in medicine, a wide range of frequencies is used, ranging from 30 kHz to 30 MHz [1,2,3]. The factors inducing hyperthermia include the following [1,4,5]:

- Long-wave diathermy—from 3 to 300 kHz (however, this is considered obsolete due to the presence of numerous practical limitations);
- Short-wave diathermy—from 3 to 30 MHz;
- Microwaves—from 300 to 3000 MHz (rarely used today).

The most widely used frequency is 27.12 MHz [6,7].

The therapeutic effect of diathermy is achieved mainly through the creation of heat. High-frequency electromagnetic waves are not able to cause the depolarization of muscles or nerves. An increase in temperature is observed due to the following:

- Changes in the orientation of the electric dipoles present in the tissue.
- The polarization of atoms and particles with the aim of creating electric dipoles.
- The movement or slip of ions and electrons in the electromagnetic field (reciprocating motion). Increases in the temperature of selected tissues during the procedure vary. The authors emphasize a close connection between temperatures and tissue vascularization as well as their level of hydration. A large amount of water ensures the proper amount of ions as well as good electrical conductivity, allowing for mechanisms responsible for an increase in temperature to take place, while the appropriate vascularization of the treatment area can cause a more rapid fall in tissue temperature directly after the procedure due to numerous blood vessels, which will allow for the removal of excess heat [8,9]. Diathermy may also be used in cases of acute conditions in which heating is not recommended. Then, non-thermal doses are used, which may be used in, for instance, swelling reduction [10].

Indiba Activ^®^ therapy, which comes from Spain, is based on capacitive–resistive electric transfer. During the procedure, the therapist has two active electrodes—resistive and capacitive. In order to carry out the procedure, it is also necessary to have a metal plate known as a “passive electrode”, thanks to which it is possible to close the electric circuit.

The capacitive electrode is covered with an insulator made of polyamide, which isolates the metal part of the electrode from the skin, causing an operation similar to the previously described capacitor. As a result, the reaction takes place mainly on the surface under the active electrode and in tissues characterized by good hydration, which increases the electrical conductivity of the tissues. In turn, the resistive electrode has direct contact with the patient’s skin during the procedure and does not have any insulator. As a result, the reaction takes place over the entire distance between the two electrodes. The greatest efficiency occurs in the area characterized by high resistivity (electrical resistance). These are elements with weaker electrical conductivity due to their lower water content, including bone tissue, cartilage, ligaments, and tendons [11,12,13].

The work of Duñabeitia et al. confirms the theory of the local activity of diathermy. The group which was subjected to therapy was made up of runners who ran recreationally. During the study, both physiological and biomechanical parameters were measured. The results showed that using a radio frequency electromagnetic field does not influence the economics of the run, VO_2_max, the respiratory exchange ratio, ventilation effectiveness, or pulse. It was emphasized that the therapy may improve muscle regeneration, which may in turn translate to an improvement in athletic results [14].

In a study conducted by Ron Clijsen et al., Doppler ultrasonography was used to determine the influence of the electromagnetic field used on the perfusion of the microcirculation of the skin as well as the intramuscular blood flow. The results show a positive influence of the activity of a resistive as well as a capacitive electrode on the microcirculation of the skin in relation to the placebo group. However, in the case of the intramuscular blood flow, positive activity is only attributed to the resistive electrode. The conclusion of the described study is the fact that the radio frequency electromagnetic field has a beneficial effect on blood flow, exclusively at the local level [15].

It must be noted that an increase in the temperature of the treatment area is directly proportional to the resistance offered by the tissues but only up to a certain moment, when too much resistance causes the complete cessation of the flow of electricity through the centers, and heat is no longer released. In such a situation, the capacitive mode is used, thanks to which electrons gather in the area of the isolator, and heat is released in locations where the charges undergo condensation. Such a situation allows for the selective activity of energy in places specified by the person performing the procedure. The selection of a resistive or capacitive mode depends on the type of condition as well as the tissue that must undergo treatment. In practice, the two electrodes are often used interchangeably during one procedure. Independent of the heating method, it is necessary to use a passive electrode, ensuring the closure of the electrical circuit [11,16,17,18,19,20].

The therapy is considered safe due to its lack of excessive heat generation between the skin and the electrode. It has properties that allow for pain reduction and an improvement in the quality of life among participants suffering from orthopedic and inflammatory conditions [7,21,22,23].

In medicine and biomedical engineering, thermography (thermovision) is used for diagnostic as well as cognitive purposes. This non-invasive method, which is devoid of any side effects for the patient and can be repeated many times over, allows the visualization of infrared radiation invisible to the human eye. The basis of thermal imaging studies is the fact that the human body is warm-blooded and, regardless of external conditions, it maintains a similar temperature to the body cavities (skull, chest, abdominal cavity, and internal organs), approximately 36.6 ± 0.7 °C. Thanks to this, it is possible to obtain information about the physiological and pathological processes occurring in the human body, which are reflected in local and global temperature changes. In the described method, the imaging uses infrared radiation in the wavelength range of 9–14 µm. It is emitted by every object with a temperature value above −273.15 °C; therefore, in the examination image, using a thermogram, the amount of energy emitted by the tissues is visible [24,25].

Currently, static thermography (TS), in which absolute temperatures are assessed, and Active Dynamic Thermography (ADT), are used to assess temperature changes on the skin surface time. This allows the thermal properties of tissues and organs to be determined. In physiotherapy, medical thermography is sometimes used as a tool to evaluate the effectiveness of physical therapy treatments. In this way, changes in skin temperature are assessed, inter alia, under the influence of cryotherapeutic procedures, with the use of ultrasounds, etc. The parameters of the stimuli are usually selected empirically or on the basis of the size of the sensory or motor reaction, hence the observation and registration of reactions occurring during and after the physical treatment. Thermovision is one of the most useful tools here, because it allows not only a one-time assessment of the effectiveness of the treatment but also allows you to track and evaluate serial treatments [26,27,28].

The aim of this retrospective work, based on the data included in a medical documentation, was to assess the dynamics of temperature changes on the body surface after the application of a high-frequency electromagnetic field depending on the type of electrode used.

## 2. Materials and Methods

### 2.1. Ethics

This retrospective study performed from February to April 2020 was conducted according to the guidelines of the Declaration of Helsinki and approved by the Institution of the Ethical Committee of the University of Technology, Academy of Silesia in Katowice no. 03/KEBN/2022 (6 May 2022). Informed consent was obtained from all subjects involved in the study.

### 2.2. Participants

The study was conducted in the Salveo Center of Active Rehabilitation in Katowice, Poland. Each participant in the study joined it voluntarily. Based on the analysis of the collected and shared documentation, out of 163 participants—physiotherapy students in the Faculty of Public Health in the Medical University of Silesia in Katowice, Poland—30 participants who met the inclusion and exclusion criteria for the study qualified for the study. In the studies conducted in April 2020, 30 healthy subjects participated (15 women and 15 men), who were physiotherapy students in the Faculty of Public Health in the Medical University of Silesia in Katowice, Poland, and they were divided into two comparative groups (A and B). In Group A, a capacitive electrode was used, while in Group B, a resistive electrode was used. The right lower limb constituted the research sample. The duration of the procedure in both groups amounted to 10 min. Table 1 presents the anthropometric data of participants.

#### Inclusion and Exclusion Criteria

The inclusion and exclusion criteria were developed based on the recommendations of The Interactive Thermology for Europe (IATE) guidelines. Eligibility for the study was based on a questionnaire, which was an integral part of the written informed consent. The questions concerned reducing to a minimum physical activity, alcohol consumption, smoking, the use of stimulants, and drugs causing changes in body temperature 7 days before the examination. On the day of examination, participants were instructed to refrain from drinking any hot or cold drinks or eating food for approximately 5 h prior. The questionnaire also asked participants to confirm their awareness of the necessity to not apply any cosmetics to the tested surface on the day of the test.

The study included participants, physiotherapy students in the Faculty of Public Health in the Medical University of Silesia in Katowice, Poland aged 20–25 who gave informed, voluntary consent to participate in the study and had no contraindications for therapy using the electromagnetic field. In addition, the criterion for inclusion in the study was to answer all the questionnaire questions confirming the fulfillment of the IATE conditions. The study included people who refrained from consuming hot or cold drinks, products and did not apply any cosmetics to the study area on the day of the study 5 h prior to the study. The study included participants who did not report chronic disease, mental disorders, pregnancy, oncological disease (now or in the past), did not have surgery in a lower limb, or did not have lower limb pain lasting more than 3 days.

The exclusion criteria from the study were the age below 20 or over 25 years of age, not being a student of physiotherapy at the Faculty of Public Health of the Medical University of Silesia in Katowice, did not provide voluntary, informed consent to the study, contraindication to electromagnetic field therapy, chronic disease, pregnancy, disease oncological (currently or in the past), mental disorders, had surgery in a lower limb, or had lower limb pain lasting more than 3 days. Participants who replied to any of the questions contained in the questionnaire indicating failure to meet the requirements of IATA guidelines were also excluded from the study. In addition, participants who, on the day of the study, did not comply with the 5-h ban on consuming hot or cold drinks before the study, and who applied the cosmetic to the study area, were not included in the study.

**Figure 1:**
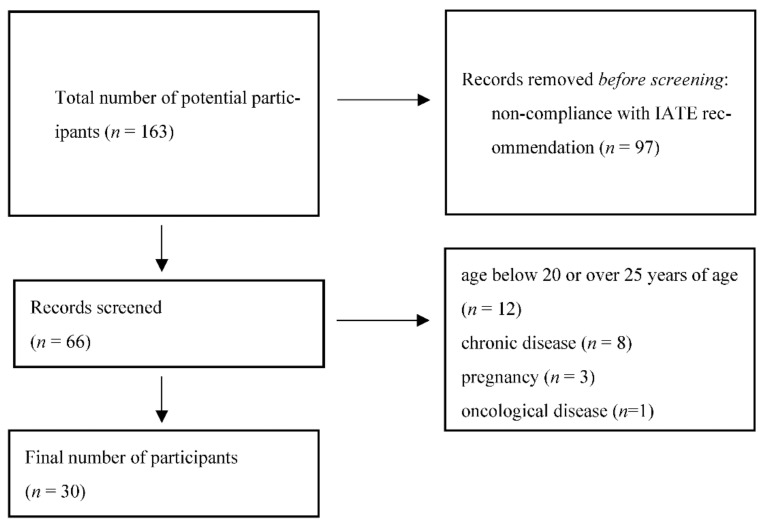
The procedure for selecting participants’ medical records that were finally analyzed.

Figure 1 shows the procedure for selecting participants’ medical records that were finally analyzed.

### 2.3. Research Room

The research room was prepared according to IATE guidelines [29,30]. It was concluded that the area of the room could not be less than 6 m^2^ (2 × 3 m), and the optimal room size was 3 × 4 m or larger. In our case, the dimensions of the room were 3 × 5 m. An important factor determining the credibility of the obtained results is maintaining the appropriate humidity of the measuring room at the level of 45–55%, which was met in this study (48.4 ± 4.1%). As recommended, there were no radiators, stoves or any kind of heater in the room. In addition, the tests were carried out with the windows closed, which were covered with a shutter to limit the light intensity as well as air circulation. There was also no air-conditioning or other devices operating in the room based on a similar principle. The recommendations as to the temperature in the room of 20–22 °C (in our study, 20.79 ± 0.3 °C) were also met. Temperature and humidity values were monitored using an electronic thermometer with a hygrometer option (Bionovo, Legnica, Poland). The accuracy of the measurement was ±1 °C; ±5%. According to the recommendations, each participant of the study was also allowed to acclimatize to the room for 60 min. During the acclimatization, the test surface was exposed in order to stabilize the heat exchange with the surroundings. The participants were also informed that they were not allowed to touch, massage or lean on them [29,30].

### 2.4. Procedures

**Figure 2:**
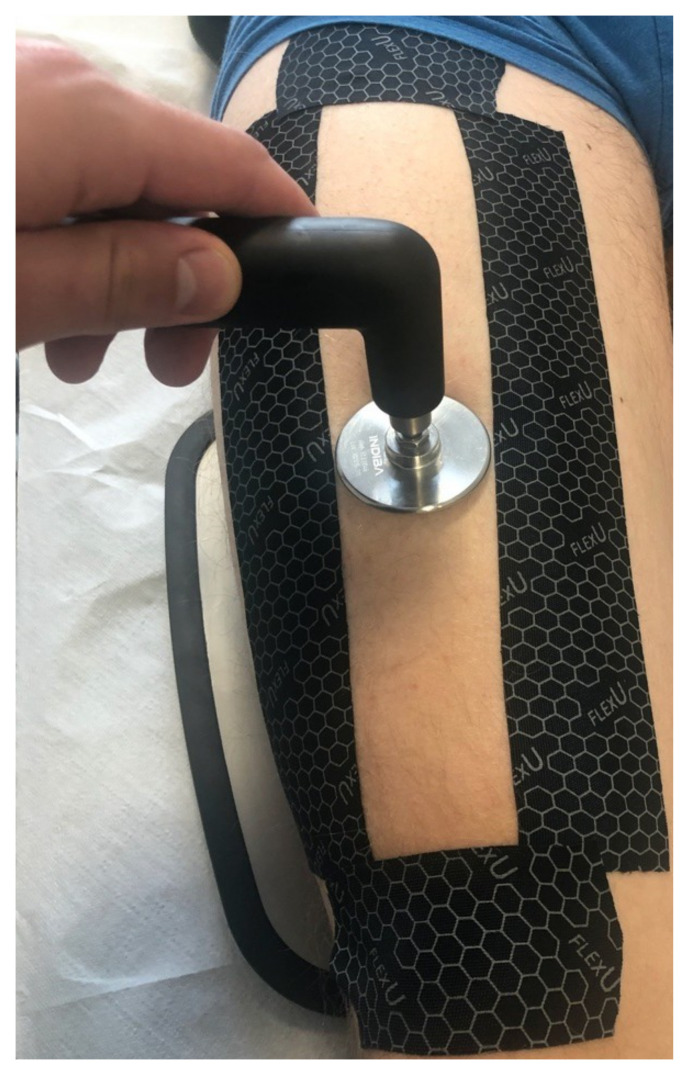
The method of determining the studied area (own picture).

**Figure 3:**
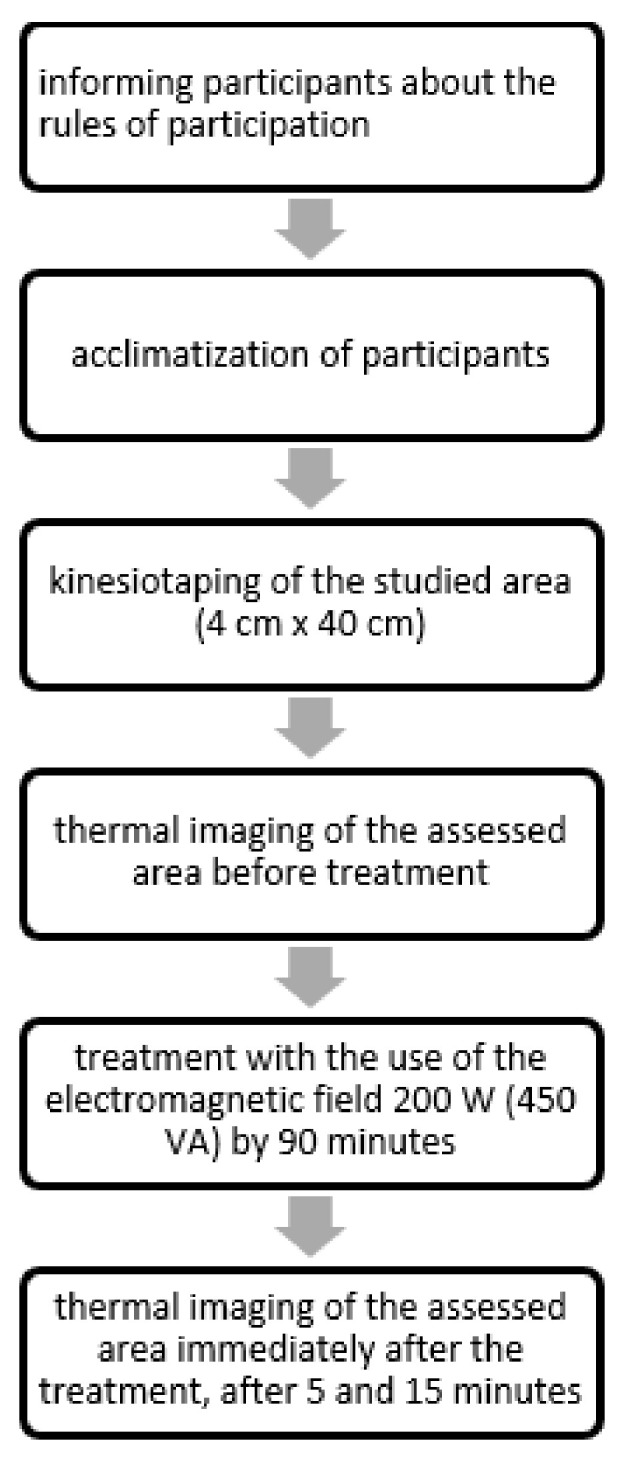
Procedure for the use of high-frequency electromagnetic field and thermal imaging of the area under examination.

The study was performed in triplicate technical repetitions for the next three weeks for each study participant. Thus, for each participant in the given observation period, three results of the tissue temperature values were obtained, totaling 12 measurements for each study participant. The area where the electromagnetic field with a radio frequency was applied was marked out with a frame made of kinesiotaping patches. Its dimensions were 4 cm wide and 40 cm long. The width of the treatment area was adjusted to the size of the head in order to limit the possibility of applying a therapeutic dose in the area that was not being tested (Figure 2). All the subjects who qualified for the procedure completed it without any discontinuation. The radio frequency electromagnetic field was well tolerated, and there were no adverse effects that could have resulted from the procedure. The strength used did not cause any thermal discomfort among the participants. The performed procedure is presented in Figure 3.

#### 2.4.1. Radio Frequency Electromagnetic Field

In order to generate a radio frequency electromagnetic field, an INDIBA ACTIV^®^ CT9 was used, with a maximum power of 200 W (450 VA). The device allows the use of a capacitive electrode and a resistive electrode with a frequency of 448 kHz.

In the study, 4 cm electrodes and a 20 × 26 cm passive electrode were used. During each procedure, 20 mL of the Prionic Activ Cream was used.

For the duration of the procedure, participants were placed in a supinated position. In the middle of the length of the hamstring muscles, a passive electrode was placed along the course of the muscle fibers. The area of the body undergoing surgery includes the area of the rectus femoris and was marked with a rectangle made out of elastic therapeutic tape. Its dimensions were 4 cm in width and 40 cm in length. The width of the treatment area was adapted to the size of the probe in order to limit the use of the therapeutic dose to only the location undergoing the procedure. The movement of the active probe was conducted along the course of the muscle fibers, while its movement was adapted to the sound of the metronome set at a frequency of 1 Hz. The probe was inserted in such a way as to not touch the plaster rectangle.

In order to perform the procedure, a strength of 155 VA (69 W) was used for the capacitive electrode and 70 VA was used for the resistive electrode (280 W), which constituted 35% of the maximum power of the device. The tested participants were informed of the need to provide feedback regarding discomfort or pain. If such symptoms were reported, the procedure was immediately discontinued. The treatment time was 15 min.

After the application, excess cream was removed with the aid of a paper towel, and a thermovision camera was used to check the temperature of the tissues.

#### 2.4.2. Thermography

In order to measure temperature, we used an HT-17 thermovision camera correlated with dedicated ThermaCAM TM Researcher Pro software, version 2.8. The equipment enabled measurements in the range of −20 °C to 300 °C, with an accuracy of ±2% or 2 °C. The device was held 50 cm away from the field and was directed at a right angle toward the treatment area. Four measurements of the temperature were taken: prior to the procedure, directly after it, and 5 and 15 min after it.

The temperature of the Regions of Interest (ROI) was determined based on a mathematical formula:

TŚRx = ∑tnn,

where tn denotes the temperature value of a given pixel, and n denotes the number of pixels constituting the designated area.

#### 2.4.3. Measurement of Body Weight, Height and Body Fat

The measurement of body weight and body fat content was performed with the use of the TANITA BC401 multifunction electronic scale. The accuracy of the measurement was ±0.1 kg for the body weight measurement and 0.1% for the proportion of adipose tissue in the body volume. The measurement of the height of the test participants was performed using a classic length meter, for which the measurement error was 0.01 m.

#### 2.4.4. Skin Moisture Measurement

The skin moisture was measured for each participant before and during the whole period of our analysis (0–15 min) using a handheld device—Corneometer R (Courage & Khazaka, Cologne, Germany). The device measures the capacity of the dielectric medium, in this case, the Stratum corneum, i.e., the upper stratum corneum of the skin. As hydration increases, its dielectric properties change. The measurement is based on the different dielectric constants of water (81) and other substances (most often <7).

The Corneometer result is given in relative units, from 0 to 130. For the measurement zones, including arms, hands, legs, and elbows, very dry skin is considered to be <35, dry is considered to be between 35 and 50, and properly moistened is considered to be >50.

The study was performed in triplicate technical repetitions for the next three weeks for each study participant. Thus, for each participant in the given observation period, three skin moisture values were obtained, totaling 12 measurements for each study participant.

### 2.5. Statistical Analysis

The test results were recorded in an Excel spreadsheet. Statistica 13.3 (Statsoft, Cracow, Poland) was used for statistical analysis. In the first stage of the statistical analysis, we used the Shapiro–Wilk test to check the normality of the distribution of our data, assuming a statistical significance threshold (*p*) of <0.05. Due to the fact that the distribution of our data was not consistent with the normal distribution, further stages of the statistical analysis were carried out with the use of non-parametric tests. Descriptive statistics methods were used to calculate the median for the variables (Me) and the lower (Q1) and upper (Q3) quartiles. In order to compare the two independent variables, a Mann–Whitney U test was used, while for more than two dependent variables, the Friedman ANOVA test was applied.

#### Size Sample Calculation

The number of physiotherapy students at the Faculty of Public Health of the Medical University of Silesia was 820. The number of participants in the study was determined using the statistical tool available at https://www.naukowiec.org/dobor.html (accessed on 1 January 2020) [31] and https://www.statystyka.az.pl/dobor/kalkulator-wielkosci-proby.php (accessed on 1 January 2020) [32]. Assuming the relative error value of 7% (as used in medical research), the required number of participants is 159. Based on the analysis of medical records, the initial number of participants was 163 (*p*-value < 0.05), and then, 30 participants were enrolled in the final analysis based on the inclusion and exclusion criteria. For a group of 820 students, the maximum error value was estimated at 18%. Therefore, assuming a *p*-value < 0.05, the required number of respondents in the study was 29 (*p*-value < 0.05).

## 3. Results

### 3.1. Changes in the Tissue Temperature Depend on the Electrode Used

**Figure 4:**
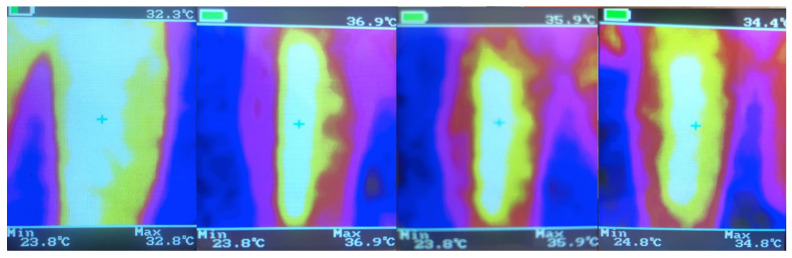
Results of measurements with a thermovision camera (own source).

The course of changes in temperature distribution on the tested surface was presented in the form of thermograms for one randomly selected participant. The remaining thermograms were similar to the example (Figure 4).

First, we assessed whether the temperature value determined at a given time of observation differed significantly between Groups A and B. It was found that these differences were not significant for the measurement carried out before using the electrode (*p* = 0.84; Mann–Whitney U test). On the other hand, at 0, 5 and 15 min, statistically significant differences were noted in the tissue temperature between the groups, depending on the electrode used (*p* < 0.05; Mann–Whitney U test). The exact results of the temperature changes between Groups A and B are presented in Table 2.

**Figure 5:**
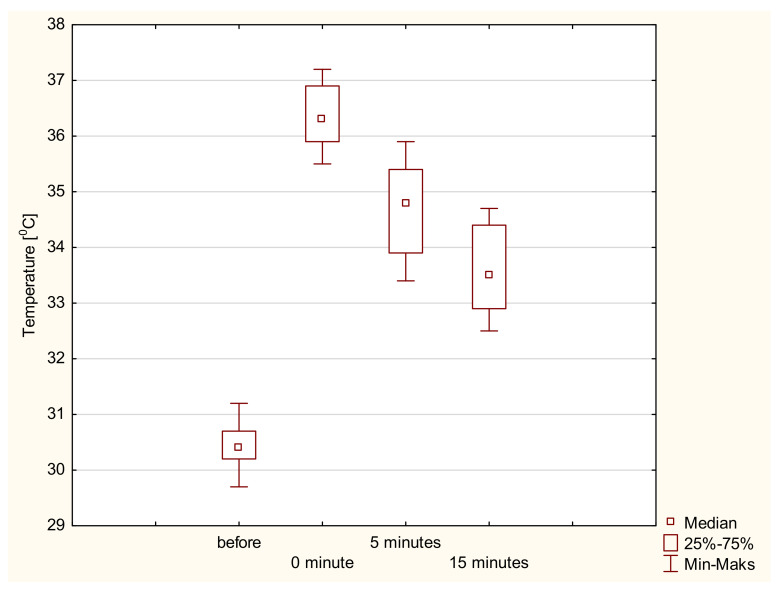
Changes in tissue temperature values as a result of using a capacitive electrode.

**Figure 6:**
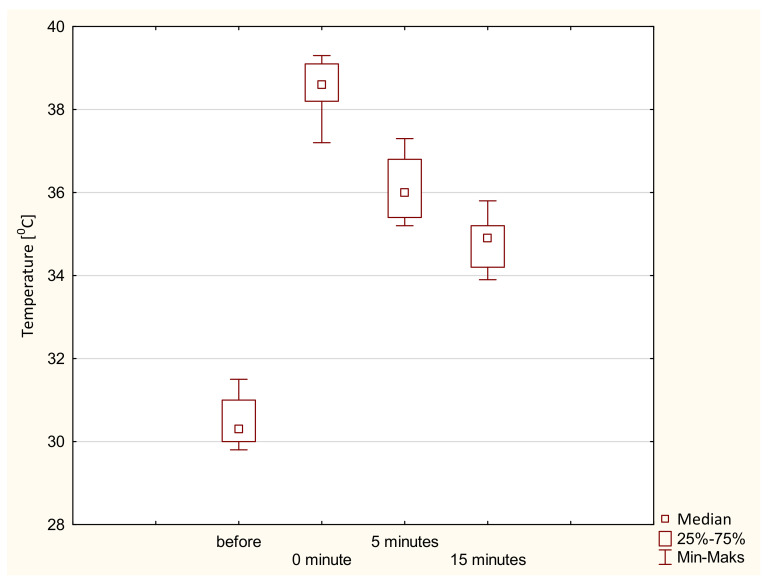
Changes in tissue temperature during the conducted observations after the use of a resistive electrode.

In the second stage, we assessed whether the changes in temperature values in Groups A and B changed statistically significantly during the observation. Based on the obtained results, it can be concluded that with the extension of the observation time, the tissue temperature increased (for Group A, Me 30.40 °C vs. 34.90 °C; for Group B, Me 30.70 °C vs. 35.20 °C). The Friedman analysis of variance (ANOVA) showed that temperature changes during the observation for both groups were statistically significant (*p* < 0.05). Therefore, in order to detail the time between which the changes were statistically significant, a post hoc Friedman ANOVA test was performed. It was shown that statistically significant differences occurred between all the observation times, regardless of the electrode used. Detailed results of the statistical analysis are presented in Table 3. Figure 5 and Figure 6 show the graphical interpretation of the results obtained for both groups depending on the type of electrode used.

### 3.2. Results of Skin Moisture

When assessing the degree of skin hydration in the participants of Groups A and B, it was noted that the skin was adequately moisturized (obtained result > 50). In both groups, there were no significant changes in skin hydration between consecutive observation times (Mann–Whitney U test), as well as between Groups A and B (Friedman ANOVA test of variance). It was noted that the median skin hydration in both groups was in the range of 64–65. Table 4 presents the results of skin moisture for Groups A and B during the observation period.

## 4. Discussion

A commonly used electrotherapy treatment that is used at high-frequency currents is Indiba^®^ therapy. The aim of the treatment is to accelerate the naturally occurring repair mechanisms of the organism.

As a result of the studies of Morelli et al., it was confirmed that despite the immediate effectiveness of procedures increasing tissue temperature on idiopathic conditions of the lumbar spine, long-lasting improvement has not been achieved [33]. This fact suggests that more studies on the effectiveness of therapies causing an increase in tissue temperature, such as the activity of a radio frequency electromagnetic field, are warranted, since using overheating without using additional physical therapy methods, such as manual therapy, medical therapeutic training, or other physical procedures, may be insufficient for complete recovery from some diseases [33,34].

In our study, we set the treatment time to 15 min, which was performed using 35% of the maximum power of the device, which was 155 VA (69 W) for a capacitive electrode and 70 VA (280 W) for a resistive electrode. This made it possible to obtain the effect of biostimulation and tissue overheating, respectively. The stimulus parameters are usually selected empirically or on the basis of the size of the sensory or motor reaction; hence, the observation and registration of reactions occurring during and after the physical treatment are particularly important in physical therapy.

On the other hand, the observation period after the Indiba^®^ treatment was selected based on the data from the literature. Yokota et al. assessed changes in temperature on the body surface in a study of 22 participants who performed intense physical exercise of the dominant lower limb and the use of capacitive and resistive electric transfer (CRET) therapy after 5, 15 and 30 min [35]. On the other hand, López-de-Celis et al. assessed changes in temperature on the surface of a superficial Achilles tendon musculotendinous junction after the application of CRET therapy in five deceased patients 1 and 5 min after the end of the treatment. In the present study, high and low power were used. When using high power, the power level of the capacitive electrode was 90 VA and 60 W for the resistive electrode. On the other hand, using the low power of the device, 20 VA for the capacitive electrode and 10 W for the resistive electrode were assumed. In conclusion, the authors concluded that the protocol with the use of high power induced a significantly greater increase in temperature on the tested surface, the effect being more pronounced for the resistive electrode than for the capacitive electrode [36]. A similar observation was also made for five deceased patients by Rodríguez-Sanz, who also assessed temperature changes 1 and 5 min after the end of the procedure [37]. The conclusions of the study by these researchers are identical to the results obtained by López-de-Celis et al. [36].

Significant factors influencing the credibility of the obtained results were the performance of the entire study including CRET therapy as well as the thermal imaging study in accordance with the recommendations of the IATE guidelines [29,30]. The application of such strict criteria is necessary because it has been clearly proven that all the above-mentioned factors have an impact on the circulatory system and thus also on the surface temperature distribution. Their effects are not only visible in the thermal image but can also last from several dozen minutes to several hours [29,30]. Thus, the influence of external factors that could affect the obtained results was limited to the absolute minimum. Petrofsky et al. assessed the effect of air temperature in the range of 38–42 °C and air humidity in the range of 0–100% on the temperature of the skin surface after a 20 min exposure to the above-mentioned conditions. The study participants comprised eight young and eight elderly people. They noticed that in the temperature range of 38–40 °C, air humidity had no significant effect on the temperature of the skin surface. On the other hand, when the participants were exposed to the temperature of 42 °C and 100% humidity, the increase in the surface temperature of the skin was the highest compared to the lower air humidity at the same ambient temperature [38].

The level of skin hydration is also important for the obtained results. Therefore, our study included the measurement of skin hydration in the area treated with Indiba^®^. The obtained results indicated the optimal level of skin hydration in this area. Observations by Park et al. indicate that the degree of skin hydration and the amount of sebum secreted correspond to changes in the temperature of the skin surface [39]. Additionally, Lu et al. indicate a relationship between the ambient temperature and the effect of a thermal stimulus (cold/heat) on the degree of skin hydration [40].

Therefore, taking into account the recommendations as well as relevant reports from the literature [29,30,38,39,40], the assessment of the surface temperature of the skin after CRET therapy, carried out in a different range of ambient temperature and humidity, would probably be interesting cognitively. It would be possible to determine the dynamics of changes in skin surface temperature under various conditions; however, they would not correspond to the conditions specified in the recommendations for thermographic tests [29,30]. The obtained results confirm that using a high-frequency electromagnetic field with the application of both kinds of overheating causes an increase in tissue temperature.

Based on our research, we noticed that regardless of the electrodes used during the Indiba^®^ treatment, there was a statistically significant increase in the temperature of the skin surface in the area where the treatment was performed (*p* < 0.05). In addition, the greatest increase in temperature was found immediately after the end of the procedure, and in the following periods of observation, the temperature gradually decreased, but it was still higher than at the beginning of the study. It is worth noting that the surface temperature of the skin before the procedure in the participants of both groups was at a similar level (Group A 30 C vs. Group B 40 C; *p* < 0.05). Therefore, the observed changes in temperature after the Indiba^®^ treatment during the observation are a direct result of the influence of the applied therapy. The obtained results indicate that the conducted therapy is characterized by a thermal effect, confirming its effectiveness [41,42,43,44].

Nevertheless, statistical analysis showed significant differences in temperature at a given point in thermography after CRET therapy depending on the electrode used. Thus, on this basis, taking into account the principle of operation of the Indba^®^ treatment and the expected effects [41,42,43,44], it can be determined which of the electrodes results in a greater temperature rise. Therefore, the conducted analysis is important for patients who do not tolerate treatments that result in an increase in body temperature. For them, it would be more desirable to undergo the Indiba^®^ treatment using a capacitive electrode. On the other hand, in patients with disturbances in temperature sensation, such as in the course of, for example, polyneuropathy [45] and cavernosa of the spinal cord, it would be necessary to perform the procedure with a resistive electrode in order to stop the patient from experiencing pain.

It was determined that an increase in tissue temperature of more than 1 °C helps in alleviating mild inflammatory conditions, and an increase of 2–3 °C allows a decrease in muscle pain and contraction, while an increase of 3–4 °C may cause changes in tissue extensibility. Increasing temperature by the above-mentioned values is known as “mild hyperthermia” in clinical practice, while in many other invasive and cytotoxic medical procedures (e.g., ablation with radio frequency), the heat is much greater [46,47,48].

Kumaran and Watson conducted a study similar to ours but on a group of 15 healthy volunteers. They also assessed the changes in the surface temperature of the skin of the shank after the Intiba^®^ treatment, depending on the electrode used. The temperature was measured immediately after the end of the treatment and 45 min after the treatment. They also noted an increase in temperature regardless of the electrode used, with a higher retention index for the resistive electrode. Nevertheless, these authors would not have noticed statistically significant differences in skin surface temperature between the groups of rollers in the 45th minute of observation [49].

Therefore, it can be assumed that an electromagnetic field with a frequency of 448 kHz causes an increase in the surface temperature of the skin and thus a short-term effect in the form of increasing the temperature in less than 45 min.

Although interesting research on CRET appeared primarily in the twentieth century, when the use of an electromagnetic field with a frequency of 448 kHz was a relatively new method, new studies on temperature changes under the influence of CRET are currently also emerging. For example, Pérez-Bellmunt et al. assessed the effectiveness of CRET on material from 10 frozen cadavers, in which they assessed the temperature of molars, incisors and skin. These researchers used the following parameters: “15 VA capacitive hypothermic (CAPH), 8 watts resistive (RES8), 20 watts resistive (RES20) and 75 VA capacitive (CAP75) were performed for 5 min each”. Based on the obtained results, it was found that the capacitive electrode increased the surface temperature of the implanted molars by 6.41 °C (*p* = 0.002) and the upper incisors with the implant by 4.71 °C (*p* = 0.001). In addition, the resistive electrode resulted in the insignificant undermining of the surface temperature of incisors without implants by 1.81 °C (*p* = 0.010) and molars with (*p* = 0.001) and without an implant (*p* = 0.008) by 4.41 °C and 3.32 °C, respectively [23]. In turn, Fousekis et al. valued the thermal reaction of the skin of the posterior part of the thigh to the therapy based on a radio frequency of 448 kHz in a postmortem sample, which was applied in the form of a standard application (Indiba^®^Activ) or combined soft tissue treatment (Indiba^®^Fascia procedure) in a total of nine healthy men (age 22 ± 3 years, body weight 75.2 ± 4.9 kg, height 178.5 ± 4.7). In conclusion, these researchers found that the application of CRET at 448 kHz can induce and maintain significant thermal adaptations of the skin, reflecting increased blood circulation and the metabolism of underlying tissues, which seems to be in line with our results [50]. Moreover, López-de-Celis also assessed the effectiveness of CRET in a postmortem sample (*n* = 10). It has been shown that CRET significantly changes the surface temperature in the range of 19.7–24% [36]. Additionally, Rodriguez-Sanz et al. showed in an increase in the surface temperature of the skin of the shoulder area after the application of CRET in a postmortem sample. The temperature was measured 1 and 5 min after the end of the treatment. A statistically significant increase in temperature was observed only 5 min after the procedure, when a high power-resistive application at the posterosuperior shoulder was used [51].

On the other hand, we showed a significant increase in temperature immediately after the procedure, which may be due to a different type of clinical material in the study.

The analgesic effect of CRET therapy is confirmed by the studies of Wachi et al., who applied it to a group of people with lower back pain (LBP). This prospective, double-blind study confirmed that CRET reduces paraspinal muscle tone in 24 and 30 subjects, respectively [52,53].

Our research has both strengths and weaknesses. The strengths of the study include that this retrospective study was conducted in accordance with the applicable research ethics frameworks. For example, in a similar study by Szurko et al., the authors state that the study was carried out in accordance with the principles of the Declaration of Helsinki, but they do not indicate that the study was approved or that consent was obtained from the relevant committees or the Polish population (the same as the population in our study) to which the study was applied. In addition, in this study, the number of participants was six, but it was not determined whether such a number of participants was representative [25]. Moreover, when analyzing the available literature, it can be noticed that studies similar to ours were conducted on a smaller number of participants [25,35,36,37,38,49,52]. Only Wachi et al. conducted research on a group of 30 participants in total, 15 in each of the two groups [53]. In addition, the entire procedure was carried out in accordance with the guidelines of thermographic studies [29,30]. Another advantage of our work is the fact that the obtained results can be applied in clinical practice thanks to their compliance with relevant guidelines [28,29], which is in accordance with other researchers who used healthy volunteers as participants in similar studies [25,45,49,51].

There are, of course, certain limitations to our study. The first and most important limitation of our study is the small size of the groups. Although, of course, based on our assessment of the sample size, the number of 30 participants is sufficient, our goal when planning future studies will be to increase the number of study participants. Second, the study we present is a single-center study. Therefore, it would be reasonable to increase the number of centers participating in the study. Third, the population was restricted to young healthy students belonging to the same university. Fourth, the study was limited by its retrospective nature. Therefore, in subsequent research, we wish to conduct a prospective study, during which it will be possible to enroll participants in a randomized manner. Fifth, it would be interesting to extend the study to perform measurements under various temperature conditions, e.g., in the range 5–35 °C. An interesting addition would be to obtain more data characterizing the study participants and to conduct multivariable analysis.

Despite the numerous factors limiting our research, taking into account its strengths as well as the fact that the topic is still relevant, our research is valuable and useful in physiotherapy practice and perhaps when taking into account the latest trends in other fields of medicine and health sciences.

## 5. Conclusions

Our study confirmed that the use of both a capacitive and resistive electrode during treatment with the use of a high-frequency electromagnetic field statistically significantly increased the surface temperature of the area to which the therapy was applied. However, a greater increase in temperature was observed for the resistive electrode. We confirmed that the thermal effect occurred immediately after the treatment. The study also provides valuable data for further research into CRET-based therapy. The results of the study can be used in clinical practice by physiotherapists to optimize the conditions of therapy. Of course, taking into account the limitations of the study, further studies are warranted, including clinical studies and the evaluation of additional physiological reactions, e.g., blood flow and deep tissue temperature.

## Figures and Tables

**Table 1:** Anthropometric data of participants.

	Group A (*n* = 15)	Group A (*n* = 15)
Sex (men; women)	7; 8	8; 7
Age (years)	24 ± 0.5	24 ± 1.1
Weight (kg)	75 ± 15.4	73.9 ± 16.5
Growth (cm)	174 ± 10.1	172 ± 7.8
BMI (kg/m^2^)	24.6 ± 4.5	24.9 ± 4.26
Fat tissue (%)	14.98 ± 2.19	15.11 ± 1.76

BMI, Body Mass Index.

**Table 2:** Tissue temperature at individual time intervals depending on the electrode used.

Group	Time	Median (°C)	Lower Quartile (°C)	Upper Quartile (°C)	*p*-Value (Mann–Whitney U Test)
A	before	30.40	30.20	30.70	0.84
B		30.30	30.00	31.00	
A	0 min	36.30	35.90	36.90	<0.00
B		38.60	38.20	39.10	
A	5 min	34.80	33.90	35.40	<0.00
B		36.00	35.40	36.80	
A	15 min	33.50	32.90	34.40	<0.00
B		34.90	34.20	35.20	

Group A, participants who used a capacitive electrode; Group B, participants who used a resistive electrode.

**Table 3:** Results of the post hoc Friedman ANOVA test for Groups A and B (*p* < 0.05).

Time	Before	0 Min	5 Min	15 Min
Before		0.00 ^1,2^	0.00 ^1,2^	0.00 ^1,2^
0 min	0.00 ^1,2^		0.00 ^1,2^	0.00 ^1,2^
5 min	0.00 ^1,2^	0.00 ^1,2^		0.00 ^1,2^
15 min	0.00 ^1,2^	0.00 ^1,2^	0.00 ^1,2^	

^1^ Results of Friedman ANOVA post hoc test for Group A, participants who used a capacitive electrode; ^2^ results of Friedman ANOVA post hoc test for Group B, participants who used a resistive electrode.

**Table 4:** The value of skin moisture in participant of Groups A and B at different observation times.

Time	Group A			Group B			*p*-Value (Mann–Whitney U Test)	*p*-Value (Friedman ANOVA Test)
	Median	Lower Quartile	Upper Quartile	Median	Lower Quartile	Upper Quartile		
**Before**	64.98	63.19	65.76	65.13	64.23	66.18	0.97 ^1^	0.96 ^1^ 0.95 ^2^
**0 min**	65.17	64.21	66.11	64.98	64.01	66.11	0.94 ^1^	
**5 min**	64.99	64.01	66.91	65.11	64.12	65.76	0.93 ^1^	
**15 min**	64.12	63.18	65.13	65.19	64.97	66.12	0.95 ^1^	

Group A, participants who used a capacitive electrode; Group B, participants who used a resistive electrode; ^1^ *p*-value obtained by Friedman ANOVA test for Group A; ^2^ *p*-value obtained by Friedman ANOVA test for Group B.

## Data Availability

The data used to support the findings of this study are included in the article.
